# Effectiveness of Pneumococcal Conjugate Vaccines Over Antibiotic-Resistant Acute Otitis Media in Children: A Systematic Review

**DOI:** 10.7759/cureus.67771

**Published:** 2024-08-25

**Authors:** Gayanthi Dissanayake, Meaza Zergaw, Mohamed Elgendy, Alvin Billey, Asra Saleem, Bushra Zeeshan, Sondos T Nassar

**Affiliations:** 1 Medicine, California Institute of Behavioral Neurosciences & Psychology, Fairfield, USA; 2 Family Medicine, California Institute of Behavioral Neurosciences & Psychology, Fairfield, USA; 3 Orthopaedics, California Institute of Behavioral Neurosciences & Psychology, Fairfield, USA; 4 Pathology and Laboratory Medicine, California Institute of Behavioral Neurosciences & Psychology, Fairfield, USA; 5 Internal Medicine, California Institute of Behavioral Neurosciences & Psychology, Fairfield, USA; 6 Dermatology, California Institute of Behavioral Neurosciences & Psychology, Fairfield, USA; 7 Dermatology, Allama Iqbal Medical College/Jinnah Hospital, Lahore, PAK; 8 Medicine and Surgery, California Institute of Behavioral Neurosciences & Psychology, Fairfield, USA

**Keywords:** streptococcus pneumoniea, serotype, antibiotic resistance (abr), pneumococcal conjugate vaccine, acute otitis media

## Abstract

One of the most prevalent childhood illnesses in the world, acute otitis media (AOM), is mainly brought on by *Streptococcus pneumoniae*, which has resulted in a significant increase in the use of antibiotics and the emergence of antibiotic-resistant (ABR) strains. The Preferred Reporting Items for Systematic Reviews and Meta-Analyses (PRISMA) 2020 criteria served as the foundation for this systematic review. We conduct a comprehensive literature search across five primary databases, including PubMed, PubMed Central, Cochrane, Science Direct, and Google Scholar, to identify eligible studies assessing the impact of pneumococcal conjugate vaccines (PCVs) on AOM incidence and ABR. Data on AOM rates, shifts in serotype distribution, and the prevalence of ABR pneumococcal strains in children under the age of 18 after PCV implementation are taken from all kinds of studies that assessed any pneumococcal conjugate vaccines (PCV 7, 10, and 13) as interventions. Eighteen records are identified as eligible for the final review. Other articles are excluded by assessing the title and abstract relevancy, applying inclusion criteria, and using critical appraisal tools. Implementing PCVs among children in the national immunization programs in most countries, particularly PCV13 has led to substantial decreases in ABR *S. pneumoniae *strains. However, serotype replacement has emerged as a challenge, with non-vaccine serotypes becoming more prevalent. Despite this, the overall burden of antibiotic resistance and AOM has decreased, underscoring the positive impact of PCVs on public health. PCVs effectively reduce the incidence of AOM and the prevalence of ABR *S. pneumoniae* in children. The vaccines play a crucial role in antibiotic stewardship by decreasing the need for broad-spectrum antibiotics. Continued surveillance and development of next-generation vaccines are essential to address serotype replacement and sustain the benefits of PCVs in combating antibiotic-resistant AOM.

## Introduction and background

Acute otitis media (AOM) is one of the leading causes of pediatric medical visits and antibiotic prescriptions worldwide between the ages of six to 24 months [1]. Around 80% of children will encounter otitis media at some point in their lives, and 80% to 90% will develop otitis media with effusion before reaching school age [2]. The condition, characterized by middle ear inflammation and infection, significantly impacts children's health, resulting in pain, fever, and potential hearing loss. Worldwide, *Streptococcus pneumoniae* (*S. pneumoniae*) (~50%), non-typeable *Haemophilus influenzae* (NTHi) (~30%), and *Moraxella catarrhalis* (*M. catarrhalis*) (~10%) are the principal causal bacterial agents of AOM [3-5]. Antibiotics are frequently used as the conventional treatment for AOM, and while they are generally effective, the issue of antibiotic resistance is becoming more and more of a problem worldwide.

Antibiotic resistance (ABR) represents a significant challenge in treating AOM [6], as resistant strains of *Streptococcus pneumoniae* and *Haemophilus influenzae* are the two most frequently observed pathogens in children with AOM [7]. They can lead to treatment failures and recurrent infections [8]. Since antimicrobials are the primary tool for treating AOM, widespread, inappropriate use of β-lactam antibiotics for AOM is thought to have contributed to the development of ABR and necessitating alternative strategies to mitigate this public health issue [9].

Pneumococcal conjugate vaccines (PCVs) have developed as a preventive measure against infections caused by *Streptococcus pneumoniae*, a common pathogen in AOM. The first polysaccharide-protein conjugate vaccine includes seven pneumococcal serotypes (PCV7). It became available in 2000 in the United States [3]. Currently, two PCVs are recommended for use in childhood immunization programs: the 10-valent (PCV10) and the 13-valent (PCV13) vaccines target multiple serotypes of the pneumococcus bacterium [10,11], aiming to reduce the incidence of invasive pneumococcal diseases, including bacteremia, meningitis, and pneumonia, along with AOM [10]. PCVs for over two decades considerably reduced the overall burden of AOM by preventing infections caused by vaccine-covered pneumococcal serotypes, with a simultaneous increase in the incidence of non-vaccine serotypes [10,12]. In addition, the herd immunity effect extends protection to non-vaccinated individuals, contributing to broader public health benefits.

Given the critical intersection between vaccine use and ABR, a systematic review evaluating the effectiveness of PCV in reducing antibiotic-resistant AOM in children is essential. Such a review will consolidate evidence on how PCVs impact the incidence and severity of AOM, specifically in the context of ABR, thereby guiding clinical practices and public health policies.

## Review

Methodology and literature search strategy

The methodology for this systematic review has been designed to ensure a comprehensive, unbiased, and transparent synthesis of available evidence on the effectiveness of PCV in reducing antibiotic-resistant AOM among children. This review is particularly relevant to researchers, healthcare professionals, and policymakers in pediatric medicine and public health. The review will adhere to the Preferred Reporting Items for Systematic Reviews and Meta-Analyses 2020 (PRISMA 2020) guidelines [13].

Eligibility Criteria

The studies were selected based on PICO T questions: Population: Children aged 0-18 years with antibiotic-resistant AOM; Intervention/Condition: Administration of any PCV (PCV7, PCV10, PCV13); Comparators: Unvaccinated groups of children with PCV; Outcomes: Effectiveness of PCV towards Incidence of AOM and antibiotic-resistant AOM, Time: 2019-2024.

Inclusion criteria comprised trials, reviews, meta-analyses, studies with a control group, and papers published in the last five years. Articles related to medicine and dentistry, biochemistry, genetics, molecular biology, pharmacology, toxicology, and pharmaceutical science were also considered. After carefully perusing titles and abstracts, only free full-text studies in English that might meet the inclusion criteria mentioned here were retrieved.

Studies published before 2019 were excluded. They involved adults and nonhuman subjects. Studies were also limited to English only. No limitations were placed on the place of publication or geographical scope of the analysis.

We conducted a comprehensive literature search (completed in May 2024) using electronic databases such as PubMed, PubMed Central, Cochrane Library, ScienceDirect, and Google Scholar. This thorough search strategy incorporates common keywords and Medical Subject Headings (MeSH) terms, depending on the database used, as shown in Table 1.

**Table 1:** The bibliographic search strategy in databases with their corresponding filters. PCV; Pneumococcal Conjugate Vaccine

Data Base	Key Words	Search Strategy	Number of Articles before filters	Filters	Number of Articles after filters
Pub Med	Otitis media, Acute Otitis Media, Antibiotic-resistant otitis media, Pneumococcal conjugate vaccine, PCV13, PCV	Acute Otitis media OR Otitis media OR antibiotic resistant Otitis media OR( "Otitis Media/classification"[Majr] OR "Otitis Media/complications"[Majr] OR "Otitis Media/diagnosis"[Majr] OR "Otitis Media/drug therapy"[Majr] OR "Otitis Media/etiology"[Majr] OR "Otitis Media/prevention and control"[Majr] ) AND Pneumococcal conjugate vaccine OR PCV13 OR PCV OR( "Pneumococcal Vaccines/adverse effects"[Majr] OR "Pneumococcal Vaccines/classification"[Majr] OR "Pneumococcal Vaccines/history"[Majr] OR "Pneumococcal Vaccines/therapeutic use"[Majr] )	12399	Inclusion: Free full text, Published within the last 5 years, Human studies, English, Children birth to 18 years old Exclusion: Studies before 2019, Animal studies, Languages other than English, Population above 18 years old	646
PMC	Acute Otitis Media, Otitis Media, , Antibiotic-resistant otitis media, Pneumococcal conjugate vaccine, PCV13, PCV	Acute Otitis Media AND Pneumococcal Conjugate Vaccine	3159	Published within the last 5 years	1082
Google Scholar	Otitis Media, Pneumococcal conjugate vaccine, PCV13, PCV, Antibiotic resistance	“Acute Otitis Media” AND “Pneumococcal Conjugate Vaccine”	8480	Published within the last 5 years, Review articles	316
Cochrane Library	Otitis Media, Antibiotic-resistant otitis media, Pneumococcal conjugate vaccine, PCV13, PCV	Acute Otitis Media AND Pneumococcal Conjugate Vaccine	104	Published within the last 5 years, English	10
ScienceDirect	Otitis Media, Antibiotic-resistant otitis media, Pneumococcal conjugate vaccine, PCV13, PCV	Acute Otitis Media AND Pneumococcal Conjugate Vaccine	2582	Published within the last 5 years, English	465

Data Collection and Screening Process

The results yielded were reviewed by two independent reviewers for their applicability, and this was done by examining the title and abstract of the records. The irrelevant papers and duplications were removed manually and by using EndNote. Full-text articles of potentially relevant studies were retrieved and assessed for eligibility by two independent reviewers. Disagreements were resolved through discussion or consultation with a third reviewer.

Data Extraction and Quality Assessment

Relevant data from the included studies was extracted systematically, and two independent individuals conducted the quality assessment. The qualities of studies were assessed using standardized tools such as the Scale for the Assessment of Narrative Review Articles 2 (SANRA 2) checklists for traditional reviews were implemented [14]. The studies attained a score of 9 or higher, included in the final results. For observational and non-randomized control trials, the Newcastle-Ottawa scale (NOS) was used [15], with studies only included if they attained a score of 7 or higher. For systematic reviews, we used AMSTAR 2, a critical appraisal tool for systematic reviews that include randomized or nonrandomized studies of healthcare interventions, or both, In which the articles with assessment scores >70% were included as high-quality studies [16]. For randomized clinical trials, the Cochrane Collaboration Risk of Bias Tool (CCRBT) was used [17], and studies that have >70% assessment scores were included with a low risk of bias in the final review.

Results

**Figure 1:**
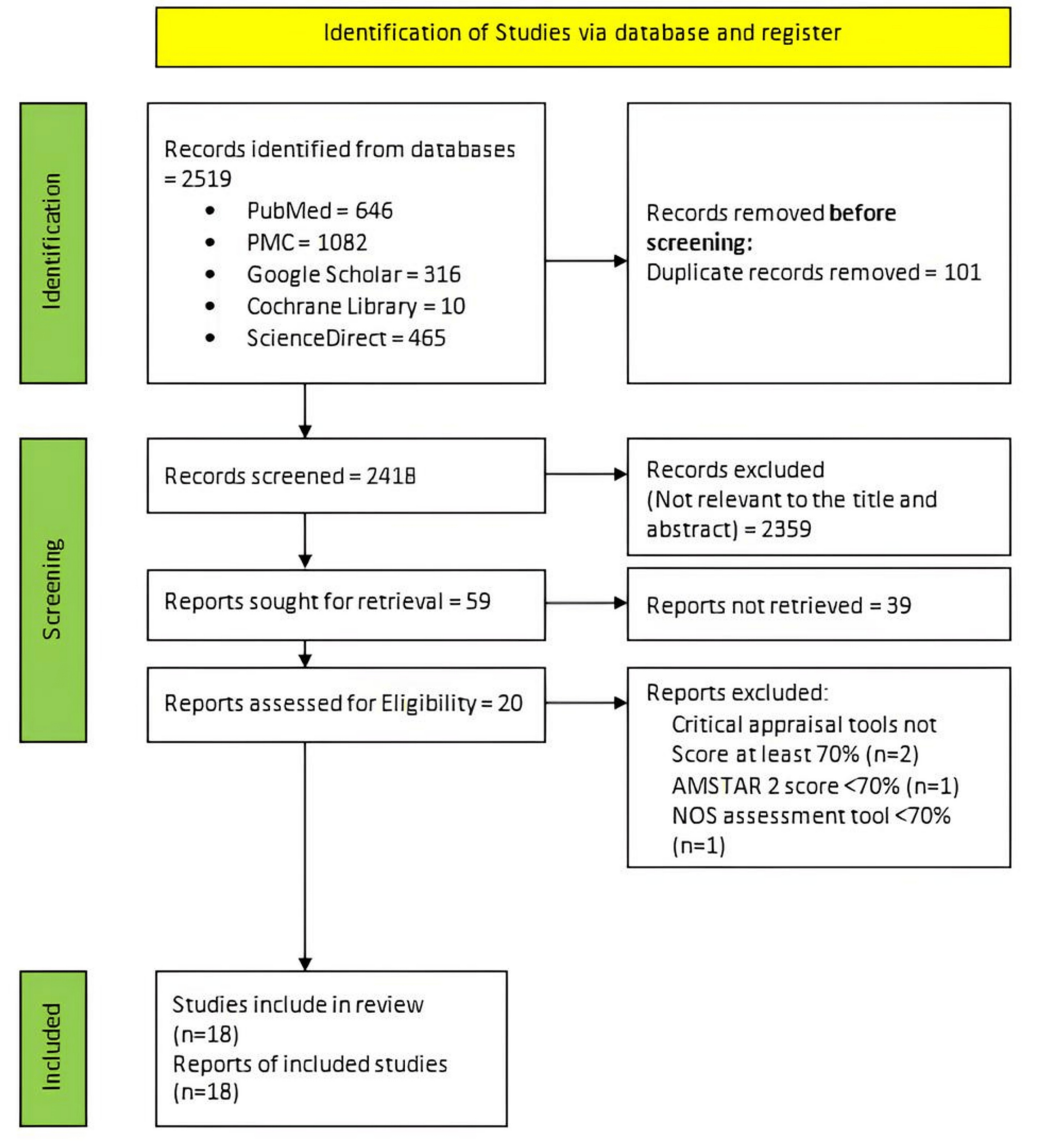
PRISMA flow diagram of the study search selection. PRISMA; Preferred Reporting Items for Systematic Reviews and Meta-Analyses [13]. PMC; PubMed Central AMSTAR; A critical appraisal tool for systematic reviews that include randomized or non-randomized studies of healthcare interventions, or both NOS; The Newcastle-Ottawa Scale

The literature search yielded 2519 records. After removing duplicates, we were left with 2418 unique records. Following a thorough title and abstract screening, 59 potentially relevant articles were identified. Unfortunately, 39 articles had to be excluded due to the unavailability of free full text, leaving us with 20 studies suitable for the quality appraisal. The first author conducted a quality assessment for the retrieved reports, which was then reviewed and agreed upon by the second and third authors, demonstrating our commitment to a collaborative and rigorous process. This led to the acceptance of 18 studies that scored more than 70% in our study. These included two narrative reviews, 10 observational studies, and six systematic reviews. In systematic reviews conducted during the last three years (2022-2024) in the same area, the investigators have excluded them from the study to reduce bias. A detailed flow diagram illustrating the screening process and study selection is presented in Figure 1*.*

Tables 2-5 show how each study was evaluated according to the corresponding study type and the final result of the evaluation process.

**Table 2:** Results of the Scale for the Assessment of Narrative Review Articles 2 (SANRA 2) assessment tool for narrative reviews by review authors. Passing score: 9/12 [14]

Author, year	Justification of the article’s importance for the readership	Statement of concrete aims or formulation of the question	Description of the literature search	Referencing	Scientific reasoning	Appropriate presentation of data	Sum	Pass/Fail
Kristen Feemster et al., 2023 [3]	2	1	1	2	2	1	9	Pass
Roxane Noharet-Koenig et al., 2023 [12]	2	2	2	2	2	2	12	Pass

**Table 3:** Results of the NOS assessment tool for observational studies by review authors. Passing score: 7/9 (The articles with assessment scores >70% were included as high-quality studies) Y; yes, N; no, N/A; not applicable NOS; Newcastle Ottawa Scale [15]

Author, Year	Representativeness of the exposed cohort	Selection of the nonexposed cohort	Ascertainment of exposure	Demonstration that the outcome of interest was not present at the start of the study	Comparability of the cohort based on design/analysis (2 points given if Confounders identified )	Assessment of the outcome	Was the follow-up long enough for the outcomes to occur	Adequacy of follow-up of cohort	Pass/Fail
Cristina Gavrilovici et al., 2022 [2]	Y	N/A	Y	N	Y	Y	Y	Y	Pass
Amaia Sánchez Arlegui et al., 2024 [4]	Y	N/A	Y	N	Y	Y	Y	N	Pass
Ravinder Kaur et al., 2021 [6]	Y	N/A	Y	Y	Y	Y	Y	Y	Pass
Ahmet Soysal et al., 2019 [8]	Y	N/A	N	N	Y	Y	Y	Y	Pass
Simon Imer Jespersen et al., 2021 [18]	Y	Y	Y	Y	Y	Y	N	N	Pass
Mark H Rozenbaum et al., 2024 [19]	Y	Y	Y	Y	Y	Y	Y	Y	Pass
N Littorin et al., 2021 [20]	Y	N/A	Y	N	Y	Y	Y	N	Pass
Sara Amari et al., 2022 [21]	Y	N/A	Y	Y	Y	Y	N	N	Pass
Mitsuyo Kawaguchiya et al., 2023 [22]	Y	N/A	N	Y	N	Y	N	N	Fail
Hannah Griffith et al., 2022 [23]	Y	N/A	Y	N	Y	Y	Y	Y	Pass
Salini Mohanty et al., 2023 [24]	Y	N/A	Y	N	Y	Y	Y	N	Pass

**Table 4:** Result summary of critical appraisal using AMSTAR 2 for systematic reviews and meta-analyses by review authors. NMA; No Meta-Analysis Performed AMSTAR 2; A critical appraisal tool for systematic reviews that include randomized or non-randomized studies of healthcare interventions, or both The articles with assessment scores >70% were included as high-quality studies [16]

Author, Year	Criterion 1	Criterion 2	Criterion 3	Criterion 4	Criterion 5	Criterion 6	Criterion 7	Criterion 8	Criterion 9	Criterion 10	Criterion 11	Criterion 12	Criterion 13	Criterion 14	Criterion 15	Criterion 16	Result
Matt Wasserman et al., 2021 [25]	Yes	Yes	No	Yes	Yes	Yes	No	Yes	No	No	NMA	NMA	No	Yes	NMA	No	Removed
Kristin Andrejko et al., 2021 [26]	Yes	Yes	Yes	Yes	Yes	Yes	Yes	Yes	No	Yes	NMA	NMA	No	Yes	NMA	Yes	Included
Niels H Holm et al., 2020 [27]	Yes	Yes	Yes	Yes	Yes	Yes	Yes	Yes	Yes	No	NMA	NMA	Yes	Yes	NMA	Yes	Included
Tiffanie Bourgeois et al., 2019 [28]	Yes	Yes	Yes	Yes	Yes	Yes	Yes	Yes	Yes	No	NMA	NMA	No	Yes	NMA	Yes	Included
T. Mark Doherty et al., 2020 [9]	Yes	Yes	Yes	Yes	Yes	Yes	Yes	Yes	Yes	Yes	NMA	NMA	Yes	Yes	NMA	Yes	Included
Li Min Wang et al., 2021 [29]	Yes	Yes	Yes	Yes	Yes	Yes	Yes	Yes	Yes	No	Yes	Yes	Yes	Yes	No	Yes	Included
Saskia Hullegie et al., 2021 [5]	Yes	Yes	Yes	Yes	Yes	Yes	Yes	Yes	Yes	No	NMA	NMA	Yes	Yes	NMA	Yes	Included

**Table 5:** Summary of quality appraisal tools used for the final 20 studies.

Quality appraisal tool	Type of Study	Number of studies	No included studies and RoB/score
Scale for the Assessment of Narrative Review Articles 2 (SANRA 2) {2}	Narrative reviews	2	2: >9/12
Assessment of Multiple Systematic Reviews (AMSTAR 2) {7}	Systematic reviews and meta-analyses	7	6: ≥70%
Newcastle Ottawa Scale (NOS) {11}	Observational Studies	11	10: ≥70%

Discussion

PCV7 was approved in 2000 and 2001 for use among children in the United States and Europe, respectively [3,12,27]. After that, PCV7 was introduced in childhood national immunization programs (NIPs) in most countries worldwide [12]. PCV10 and PCV13 with broader serotype coverage were licensed in 2009 and 2010 respectively, and also gradually replaced PCV7 from childhood immunization programs in all countries [3,12,27]. PCV15 was approved in the US and the European Union (EU) for use in infants and children ≥six weeks of age in 2022. In 2023 PCV20 was also approved in the US in infants ≥six weeks of age but it is currently under investigation for use in infants in the EU [3].

PCVs have demonstrated significant efficacy in reducing the incidence of AOM [6]. For example, Soysal et al. (2019) found that the PCV7 vaccine reduced the number of episodes of acute otitis media from any cause by 6%, culture-confirmed pneumococcal episodes by 34%, and the number of episodes due to the serotypes contained in the vaccine by 57% [8]. In the United States of America, after introducing PCV7, the incidence of otitis media has reduced by 28%, and the antimicrobial prescriptions decreased by 42% [2,9]. A further 19% reduction happened after PCV7 was replaced by PCV13 which includes additional serotype coverage [2,27]. In Sweden, the incidence of severe OM in children younger than two years old decreased by 32% after the introduction of PCV13 [2]. This decline in AOM cases is critical as it directly correlates with decreased antibiotic use [29], thereby reducing the selection pressure for antibiotic-resistant strains.

Reduction in Antibiotic-Resistant Strains

The emergence of a serotype 19A strain colonizing the nasopharynx and causing AOM infections in primary care settings in early 2007 exhibited resistance to all US Food and Drug Administration-approved antibiotics for children.

**Figure 2:**
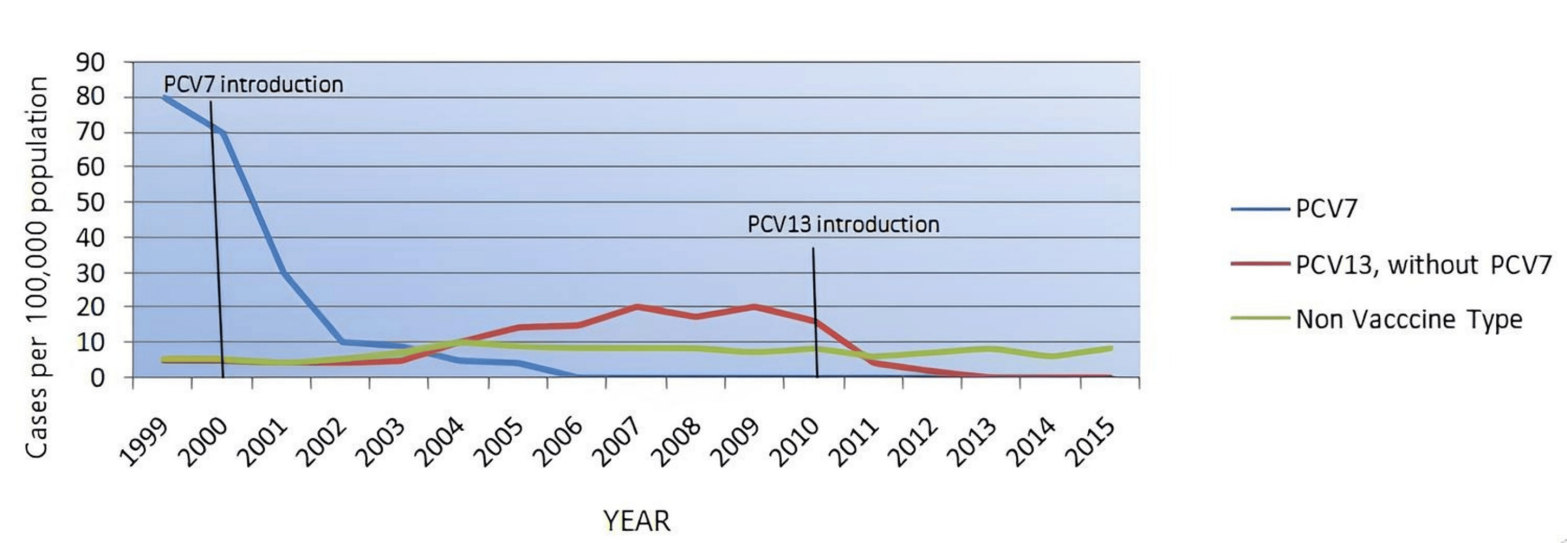
Incidence of acute otitis media (AOM) cases over 15 years after the introduction of PCV7 and PCV13 PCV; Pneumococcal Conjugate Vaccine Data compiled from study [8].

The effectiveness of PCVs in reducing antibiotic-resistant AOM is evident in several studies. Penicillin non-susceptible pneumococci (PNSP) are associated with non-vaccine serotypes (NVT) [21], and also it is usually 27.5% among AOM children [21]. Kaur et al. (2021) conducted a prospective study showing that PCV7 implementation significantly decreased the prevalence of penicillin-resistant *S. pneumoniae* and decreased resistance after the initial Introduction of PCV7 was described for macrolides and fluoroquinolones [6,29]. Littorin et al. (2021), a retrospective study, also found the results of decreased prevalence of *S. pneumoniae* isolated with *M. catarrhalis* combination by 67% after PCV introduction [20]. The broader coverage provided by PCV13 has further enhanced this effect with the near elimination of serotype 19A nasopharyngeal colonization and infections, significantly decreasing penicillin resistance among pneumococcal strains [5]. This reduction is critical because it limits the pool of antibiotic-resistant bacteria [23], making infections easier to treat with standard antibiotics. Figure 2 illustrates the changes in AOM cases over 15 years after the introduction of PCVs.

Serotype Replacement 

**Figure 3:**
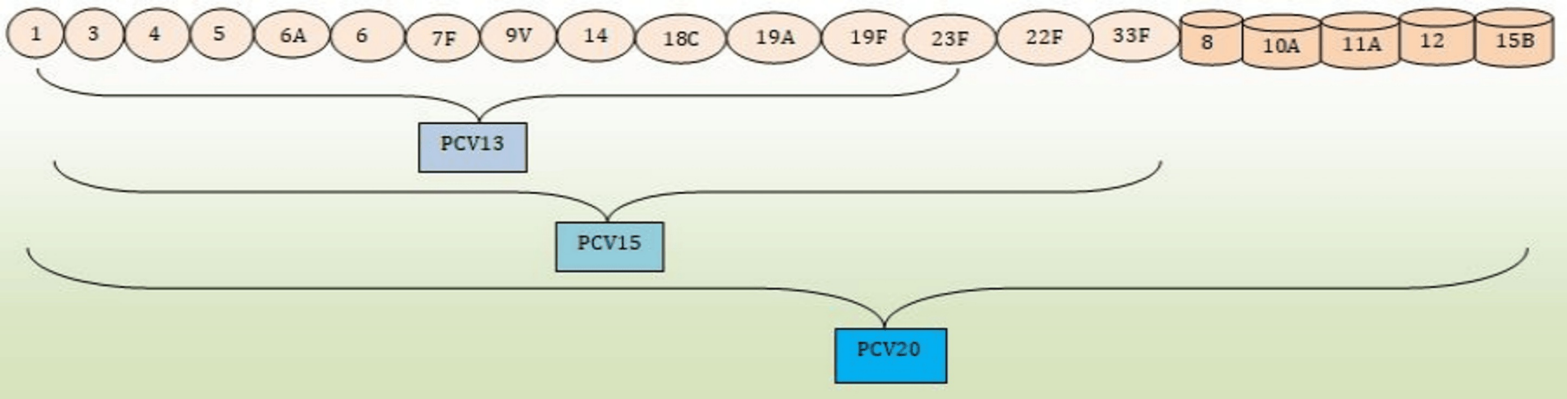
Serotype coverage by different Pneumococcal Conjugate Vaccines (PCVs) Data compiled from studies [3] and [21].

One of the unintended consequences of widespread PCV use is serotype replacement [21]. This phenomenon occurs when NVT colonize the nasopharynx and cause infections instead of vaccine-covered serotypes (VT). According to Amari et al. (2022), while there is a relative drop in VTs isolated from the middle ear specimens, a significant increase in the prevalence of NVT is demonstrated after the wide use of PCV [21]. These NVTs can still cause AOM, and the overall burden of the disease may remain reduced if these serotypes are less virulent or less likely to be antibiotic-resistant [24]. However, ongoing surveillance is essential to monitor the potential emergence of antibiotic resistance among these NVTs. Serotype coverage by different PCVs is elaborated in Figure 3.

Clinical Implications

The shift in serotype distribution necessitates continual adaptation of vaccine formulations. Next-generation PCVs, such as the 15-valent (PCV15) and 20-valent (PCV20) vaccines [12], are being developed to cover more serotypes, including those that have emerged as significant pathogens post-PCV introduction. These efforts aim to maintain and enhance the protective effects of vaccination against both AOM and antibiotic-resistant infections.

Antibiotic Stewardship and Public Health

First-line treatment in most patients with AOM is high-dose amoxicillin (80-90 mg/kg/day in two divided doses) or amoxicillin-clavulanate (90 mg/kg/day of amoxicillin with 6.4 mg/kg/day of clavulanate in two divided doses) [4,28]. Amoxicillin is recommended as a first line because of its efficacy against common AOM bacterial pathogens, safety, affordability, acceptable taste, and narrow microbiologic spectrum [28]. Other treatments for AOM in penicillin-allergic patients are oral cefdinir, cefuroxime, cefpodoxime, or ceftriaxone, administered intramuscularly [28]. Alternative treatment options for antibiotic failure after 48-72 hours include ceftriaxone or clindamycin, with or without a third-generation cephalosporin, tympanocentesis, with or without a specialist consult. The standard duration of therapy for AOM is unclear, but the recommendation is for 10 days. However, a course of treatment for five to seven days may be adequate [28].

PCVs have positively impacted antibiotic stewardship [8,9]. Most of the randomized clinical trials and prospective studies included in Doherty et al. (2020) showed that Pneumococcus-vaccinated cohorts have a significant overall reduction in antimicrobial prescribing in the range of 5-17% [9]. For otitis media, there is a sharp decline in antimicrobial use from 21% to 73% [9]. When classified by age, children under 36 months saw 20% reductions in prescribing antibiotics for OM [9]. This reduction helps preserve existing antibiotics' effectiveness and limits the spread of resistant strains.

Although antimicrobial resistance has emerged as a global public health threat and a leading cause of death worldwide [19], vaccines have been shown to mitigate drug-resistant* S. pneumoniae* infections, and vaccination is considered a critical component in the battle against antimicrobial resistance [19]. However, the Increasing rate of penicillin-non-susceptible *Streptococcus pneumoniae* may be the main cause of failure of antibiotic treatment in AOM children, and there is a recent increase in antimicrobial-resistant S. pneumoniae due to serotypes not covered by PCV13 [24].

Economic and Healthcare Burden

Fewer AOM cases and reduced antibiotic resistance translate to significant economic benefits [19]. Decreased healthcare visits, fewer complications, and lower antibiotic costs contribute to healthcare savings. Furthermore, the societal benefits of reduced transmission of antibiotic-resistant bacteria underscore the importance of widespread PCV immunization for public health.

Global Disparities and Challenges: Vaccine Coverage and Continued Surveillance

Despite the proven benefits of PCVs, global disparities in vaccine coverage remain challenging [29]. Low- and middle-income countries often face barriers to vaccine access, resulting in lower immunization rates and higher burdens of antibiotic-resistant AOM [29]. International initiatives, such as the Gavi Alliance, aim to address these disparities by increasing vaccine availability and affordability in these regions.

Despite the success of pneumococcal vaccination programs, it's essential to acknowledge that vaccine breakthroughs and failures still occur in some developing parts of the world. These instances result in a substantial global burden, particularly among the most vulnerable and the pediatric population, leading to high morbidity and mortality [3]. This underscores the need for continued research and development in pneumococcal vaccination.

Ongoing surveillance of pneumococcal serotypes and resistance patterns is crucial for informing vaccine policy and development [26]. Comprehensive monitoring systems are necessary to track changes in serotype prevalence, antibiotic resistance trends, and the overall impact of vaccination programs [26]. This data is vital for guiding the evolution of PCVs and ensuring their continued effectiveness.

Development of New Vaccines

Some studies have shown that children who were entirely and timely vaccinated with PCV13 did not have a reduced number of AOM episodes compared to children who were not vaccinated promptly [18]. Research is ongoing to develop vaccines that cover an even broader range of pneumococcal serotypes.

New higher-valent next-generation pneumococcal vaccines, such as PCV15 and PCV20, were recently evaluated and designed to address the issue of serotype replacement and provide enhanced protection against emerging serotypes in Europe and the United States [12]. These vaccines promise further to reduce the incidence of AOM and antibiotic-resistant infections and prevent more invasive cases and deaths [12].

Further studies also need to explore differences in antibiotic prescribing patterns for AOM, as changes in antibiotic prescribing could directly affect the management of recurrent OM episodes, the prevention of complications, and the development and spread of antimicrobial resistance.

## Conclusions

PCVs have proven highly effective in reducing the incidence of AOM and the prevalence of antibiotic-resistant *S. pneumoniae* in children. By targeting vital pneumococcal serotypes, PCVs decrease the overall burden of AOM and play a critical role in antibiotic stewardship. The reduction in antibiotic-resistant strains and the benefits of herd immunity highlight the importance of maintaining high vaccination coverage worldwide. While challenges such as serotype replacement and global disparities in vaccine access remain, ongoing research and public health initiatives aim to address these issues. The continued evolution and implementation of PCVs represent a vital component in the fight against antibiotic-resistant infections and in improving pediatric health outcomes worldwide.
